# Influence of Test Specimen Geometry and Water Soaking on the In Vitro Cytotoxicity of Orthocryl^®^, Orthocryl^®^ LC, Loctite^®^ EA 9483 and Polypropylene

**DOI:** 10.3390/molecules27123949

**Published:** 2022-06-20

**Authors:** Richard Behnke, Franka Stahl, Kathrin Duske, Mareike Warkentin, Margit Schwartz, Burkhard Hinz, Udo Walther

**Affiliations:** 1 Department of Orthodontics, University Dental School, Rostock University Medical Center, 18057 Rostock, Germany; richard.behnke@med.uni-rostock.de (R.B.); franka.stahl@med.uni-rostock.de (F.S.); kathrin.duske@med.uni-rostock.de (K.D.); 2 Department of Materials Science and Medical Engineering, University of Rostock, 18119 Rostock, Germany; mareike.warkentin@uni-rostock.de; 3 Institute of Pharmacology and Toxicology, Rostock University Medical Center, 18057 Rostock, Germany; margit.schwartz@med.uni-rostock.de (M.S.); udo.walther@med.uni-rostock.de (U.W.)

**Keywords:** orthodontics, removable appliances, resins, polymers, monomer release, cytotoxicity

## Abstract

Depending on their composition, plastics have a cytotoxic potential that needs to be evaluated before they are used in dentistry, e.g., as orthodontic removable appliances. Relevant guidelines set out requirements that a potential new resin in the medical field must meet, with a wide scope for experimental design. In the present study, test specimens of different geometries consisting of varying polymers (Orthocryl^®^, Orthocryl^®^ LC, Loctite^®^ EA 9483, Polypropylene) were soaked for different periods of time, then transferred to cell culture medium for 24 h, which was subsequently used for 24-h cultivation of A549 cells, followed by cytotoxicity assays (WST-1, Annexin V-FITC-propidium iodide (PI) flow cytometry). In this context, a reduction in the cytotoxic effect of the eluates of test specimens prepared from Orthocryl^®^ LC and Loctite^®^ EA 9483 was particularly evident in the Annexin V-FITC-PI assay when the soaking time was extended to 48 h and 168 h, respectively. Consistent with this, a reduced release of potentially toxic monomers into the cell culture medium, as measured by gas chromatography-mass spectrometry, was observed when the prior soaking time of test specimens of all geometries was extended. Remarkably, a significant increase in cytotoxic effect was observed in the WST-1 assay, which was accompanied by a higher release of monomers when the thickness of the test sample was increased from 0.5 to 1.0 mm, although an elution volume adapted to the surface area was used. However, further increasing the thickness to 3.0 mm did not lead to an increase in the observed cytotoxicity or monomer release. Test specimens made of polypropylene showed no toxicity under all test specimen sizes and soaking time conditions. Overall, it is recommended to perform toxicity studies of test specimens using different geometries and soaking times. Thereby, the influence of the different specimen thicknesses should also be considered. Finally, an extension of the test protocols proposed in ISO 10993-5:2009 should be considered, e.g., by flow cytometry or monomer analysis as well as fixed soaking times.

## 1. Introduction

Plastics have been established as materials in medicine over a long period of time. In dentistry, they are an indispensable basis for removable orthodontic appliances, but they are also used in prosthetics and many other areas of dentistry. The most important material here is polymethyl methacrylate (PMMA) [1]. Parallel to the use of PMMA, alternative resins have been developed. In this context, light-curing resins are considered to have a lower allergy potential than PMMA due to their composition [2,3,4,5,6,7,8,9,10]. Nevertheless, the search for further alternatives is important, as all previous resins have a certain allergenic and partly toxic potential [3,8,11,12,13,14,15]. For example, certain material properties, such as high hardness and castability [16] make epoxy resins promising alternatives to the plastics currently in use. In the medical field, they are already used as a material for attaching electrodes to pacemakers or as their embedding material [17]. Polypropylene, which belongs to the group of polyolefins, is also a potential candidate for the production of orthodontic appliances by injection molding due to its sterilizability, high melting point, and low tendency to form stress cracks [18]. In addition, polypropylene is more biocompatible compared to other polymer materials [19].

The basis for in vitro testing of biological compatibility is provided by ISO 10993-5:2009 (Biological evaluation of medical devices–Part 5: Tests for in vitro cytotoxicity) [20]. This guideline suggests the use of some possible assays but does not specify exact cell lines and watering intervals prior to testing the materials. Following this guideline, a quantitative assessment of possible cytotoxic effects should be performed. Thereby, cytotoxicity can be assumed as a reduction of cell viability by more than 30%, independent of the assay or cell line used. Test specimen geometries for cytotoxicity testing are also not defined in ISO 10993-12:2021 [21] and consequently vary in previous publications. For example, test specimens with hexagonal, round, and different shapes, as well as thicknesses ranging from 0.4 to 2.5 mm and diameters ranging from 5.0 mm to 50.0 mm, have been utilized for testing dental materials [3,8,13,22,23,24,25,26]. Some authors also used fragmented specimens [13,22]. Moreover, the surface area to extraction volume ratio is not considered in most publications. In terms of soaking time, in addition to 48 h, a longer time of 168 h or no soaking was investigated [3,8,13]. Similarly, a wide variety of cell types, from gingival fibroblasts to cervical cancer cells (HeLa), as well as different cytotoxicity assays, such as the MTT (3-(4,5-dimethylthiazol-2-yl)-2,5-diphenyltetrazolium bromide) test, agar overlay assay or flow cytometry were used [3,8,13,14,22,23,24,25,26].

**Figure 1:**
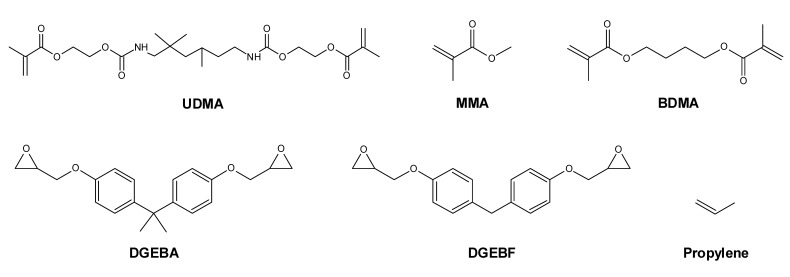
Structural formulas of monomers, which are the basis for the resins in the present study: urethane dimethacrylate (UDMA), methyl methacrylate (MMA), 1,4-butanediol dimethacrylate (BDMA), bisphenol A diglycidyl ether (DGEBA), bisphenol F diglycidyl ether (DGEBF) and propylene (propene).

The aim of this study was to determine the influence of sample size and soaking time on the cytotoxicity of and monomer release from plastics. Most resins include a mixture of monomers, which can have cytotoxic effects [3,8,25,26,27]. For this purpose, test specimens of different sizes were tested while maintaining a constant ratio of surface area to extraction volume in order to achieve comparable monomer release. Various plastics were selected for these studies. Thus, in addition to the cold-curing PMMA polymer of Orthocryl^®^ and the light-curing resin Orthocryl^®^ LC, the epoxy resin Loctite^®^ EA 9483 and a polypropylene test specimen were investigated for their potential cytotoxic properties and their monomer release after a soaking time of up to 168 h. In Figure 1 the structural formulas of the monomers are shown, which are the basis for the resins in this study. Experiments were performed on A549 cells, which are a widely used model for cytotoxicity studies of dental materials [28,29,30].

## 2. Results

### 2.1. Experimental Setup

For the investigation of cytotoxicity and the measurement of leaked monomers, test specimens (each diameter × thickness): A, 15 mm × 0.5 mm; B, 50 mm × 1.0 mm; C, 50 mm × 3.0 mm) were prepared according to clinically relevant sizes and stored in distilled water (soaking) for different time intervals (0, 48 or 168 h). The corresponding test specimens were made of Orthocryl^®^, Orthocryl^®^ LC, Loctite^®^ EA 9483 and polypropylene, respectively. After the indicated watering times, the test specimens were placed in a cell culture medium for 24 h. Finally, the same culture medium (eluates) was used undiluted (100%) or at different dilutions (50%, 25%, 12.5%, 6.25%, 3.125%) for the 24 h cultivation of A549 cells. Pure culture medium that had not previously been in contact with the test specimens was used as a control. Subsequently, the cytotoxicity of the respective eluates was determined by viability assay (WST-1 assay) and flow cytometry (Annexin V-FITC-propidium iodide (PI) assay). Monomers were determined in undiluted eluates.

According to ISO 10993-5:2009 [20], a possible cytotoxic effect is to be assumed beginning with a 30% decrease in cell viability, which was partially observed in our tests. In the figures and results texts below, the number following the single resin describes the dilution of the undiluted extract with culture medium, e.g., Orthocryl^®^ 25 corresponds to a concentration of 25% of the original undiluted extract.

### 2.2. Influence of Eluates from Test Specimens on Cellular Viability According to the WST-1 Assay

**Figure 2:**
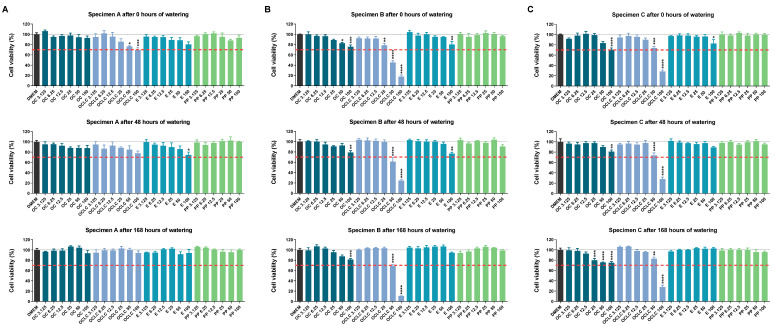
Viability of A549 cells measured by WST-1 assay after incubation with eluates from (**A**) test specimens A (15 mm × 0.5 mm), (**B**) test specimens B (50 mm × 1.0 mm) or (**C**) test specimens C (50 mm × 3.0 mm) of different compositions (OC, Orthocryl^®^; OCLC, Orthocryl^®^ LC; E, Loctite^®^ EA 9483; PP, polypropylene) and previous watering conditions. The test specimens were stored in distilled water at room temperature for 0, 48 or 168 h followed by a 24 h storage in cell culture medium (DMEM, Dulbecco’s Modified Eagle Medium) under shaking conditions. The DMEM eluates were used undiluted (100%) or diluted (50, 25, 12.5, 6.25, 3.125%) for a 24 h incubation of A549 cells followed by determination of cell viability using the WST-1 assay. Percentages represent comparison with cells treated with DMEM vehicle control (set to 100%). The data are mean values ± SEM of *n* = 3–4 per group. * *p* ≤ 0.05, ** *p* ≤ 0.01, *** *p* ≤ 0.001, **** *p* ≤ 0.0001 vs. DMEM control; one-way ANOVA with Dunnett’s post hoc test. The dashed red line shows the 30% deviation of the treatment groups from the control value (DMEM group).

According to Figure 2 different effects on cellular responses were observed depending on the geometry of the test specimen. As expected, no negative effects of polypropylene on cells were observed, regardless of the geometry of the test specimen and the duration of soaking.

For test specimen geometry A (Figure 2A), Orthocryl^®^ also showed no significant cytotoxic effects. In contrast, significantly lower viability was detected for Orthocryl^®^ LC in undiluted extracts after 0 h, whereas the corresponding effect was only attenuated after 48 h of soaking and disappeared after 168 h of soaking. In the case of Loctite^®^ EA 9483, reduced viability was detected for the undiluted eluate after 0 h and 48 h of soaking, which was also no longer evident after 168 h of soaking. With reference to the 30% range defined in the ISO 10993-5:2009 [20] guideline, only the average Orthocryl^®^ LC 100 value was above this limit (Figure 2A).

In contrast to test specimen A, the test specimens of geometry B (Figure 2B) and C (Figure 2C) caused comparatively stronger decreases in the viability of the cells. In particular, the cells reacted to the eluates of Orthocryl^®^ LC and Loctite^®^ EA 9483 with reduced viability. Thereby, the 30% difference versus control was exceeded for test specimen B by the eluates Orthocryl^®^ LC 100 and 50 (all durations of watering) and for test specimen C by the eluates Orthocryl^®^ LC 100 (all durations of watering) and Orthocryl^®^ 100 (no watering). For specimen B, a considerable protective effect of soaking was observed for Orthocryl^®^ LC 25 and 50, but not for the respective undiluted extract. In the case of test specimen C, corresponding effects were evident in the case of Orthocryl^®^ LC 50. Interestingly, cell viability decreased measurably after 168 h of soaking Orthocryl^®^ test specimens, but within the 30% range.

As required by ISO 10993-5:2009 [20], extract concentrations of less than 25% were also tested. However, these eluates (12.5%, 6.25%, 3.125%) showed no effect on cell viability and were, therefore, not included in further experiments.

### 2.3. Influence of Eluates from Test Specimens on Cellular Viability and Apoptosis According to the Annexin V-FITC-PI Assay

Of the proportions of different cell populations determined by FACS analysis, the quantitatively most prominent and thus meaningful proportions of living and early apoptotic cells should be presented below.

**Figure 3:**
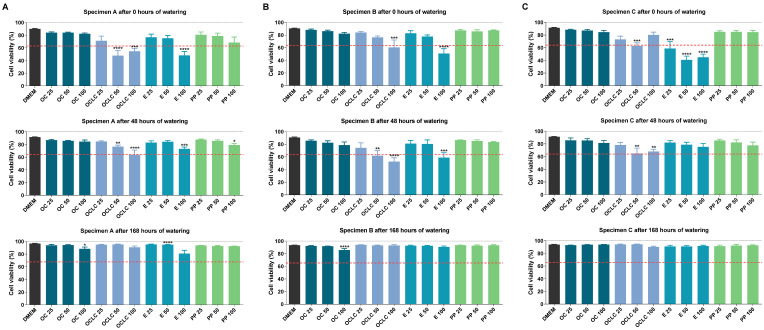
Viability of A549 cells measured by Annexin V-FITC-PI assay after incubation with eluates from (**A**) test specimens A (15 mm × 0.5 mm), (**B**) test specimens B (50 mm × 1.0 mm) or (**C**) test specimens C (50 mm × 3.0 mm) of different compositions (OC, Orthocryl^®^; OCLC, Orthocryl^®^ LC; E, Loctite^®^ EA 9483; PP, polypropylene) and previous watering conditions. The test specimens were stored in distilled water at room temperature for 0, 48 or 168 h followed by a 24 h storage in cell culture medium (DMEM, Dulbecco’s Modified Eagle Medium) under shaking conditions. The DMEM eluates were used undiluted (100%) or diluted (50 and 25%) for a 24 h incubation of A549 cells followed by determination of cell viability using the Annexin V-FITC-PI assay. Percentages refer to the sum of all cell populations measured in FACS analysis per treatment group (set to 100%). The data are mean values ± SEM of *n* = 3–4 (**A**) or *n* = 4 (**B**,**C**) per group. * *p* ≤ 0.05, ** *p* ≤ 0.01, *** *p* ≤ 0.001, **** *p* ≤ 0.0001 vs. DMEM control; one-way ANOVA with Dunnett’s post hoc test. The dashed red line shows the 30% deviation of the treatment groups from the control value (DMEM group).

**Figure 4:**
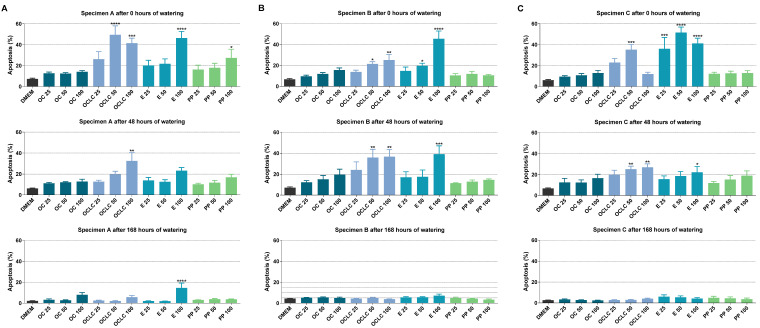
Early apoptosis of A549 cells measured by Annexin V-FITC-PI assay after incubation with eluates from (**A**) test specimens A (15 mm × 0.5 mm), (**B**) test specimens B (50 mm × 1.0 mm) or (**C**) test specimens C (50 mm × 3.0 mm) of different compositions (OC, Orthocryl^®^; OCLC, Orthocryl^®^ LC; E, Loctite^®^ EA 9483; PP, polypropylene) and previous watering conditions. The test specimens were stored in distilled water at room temperature for 0, 48 or 168 h followed by a 24 h storage in cell culture medium (DMEM, Dulbecco’s Modified Eagle Medium) under shaking conditions. The DMEM eluates were used undiluted (100%) or diluted (50 and 25%) for a 24 h incubation of A549 cells followed by determination of cell viability using the Annexin V-FITC-PI assay. Percentages refer to the sum of all cell populations measured in FACS analysis per treatment group (set to 100%). The data are mean values ± SEM of *n* = 4 per group. * *p* ≤ 0.05, ** *p* ≤ 0.01, *** *p* ≤ 0.001, **** *p* ≤ 0.0001 vs. DMEM control; one-way ANOVA with Dunnett’s post hoc test.

For test specimen A (Figure 3A), measurable viability losses for Orthocryl^®^ LC 100 and 50 were detected in accordance with the WST-1 data, although these no longer occurred after 168 h of soaking. Similarly, soaking resulted in a time-dependent decrease in toxicity caused by Loctite^®^ EA 9483 100. Consistent with this, soaking also led to a marked decline in the significantly increased number of apoptotic cells recorded in the presence of Loctite^®^ EA 9483 50, Loctite^®^ EA 9483 100 and Orthocryl^®^ LC 100 (Figure 4A). Interestingly, significant apoptosis induction could also be detected for polypropylene 100 prior to soaking, which, however, dropped to vehicle control level during 168 h soaking.

The proportion of living cells after treatment with eluates of test specimen B (Figure 3B) and test specimen C (Figure 3C) essentially corresponded to the results of test specimen A. Again, no viability-reducing effects of Orthocryl^®^ LC and Loctite^®^ EA 9483 could be detected after 168 h of soaking. In the absence of soaking, the corresponding viability reductions in the case of Loctite^®^ EA 9483 were comparatively more pronounced in test specimen C than in test specimen B. A similar pattern could also be demonstrated at the level of the apoptotic cells (comp. Figure 4B,C). Here, too, the proportions of early apoptotic cells were at the vehicle control level after 168 h of soaking.

In the synopsis of the FACS results, all visible or significant changes in the number of viable and early apoptotic cells disappeared after a soaking time of 168 h, regardless of geometry. At this point, the FACS assay differs from the WST-1 test, in which a clear loss of viability by Orthocryl^®^ LC 100 was still registered after 168 h of soaking when test specimen geometries B and C were used.

### 2.4. GC-MS Analysis of Monomer Release from Test Specimens

**Figure 5:**
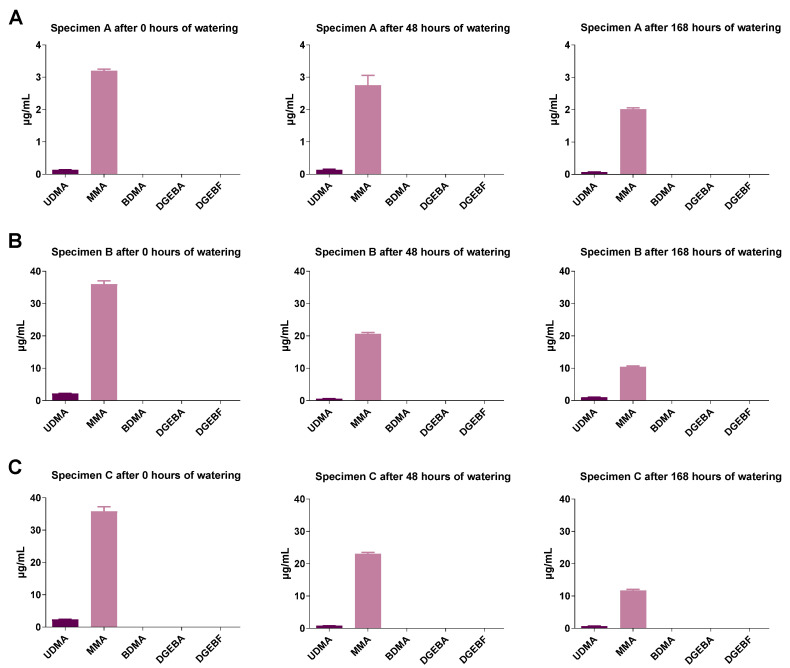
Monomer concentrations in Orthocryl^®^ specimen extracts of (**A**) test specimens A (15 mm × 0.5 mm), (**B**) test specimens B (50 mm × 1.0 mm) or (**C**) test specimens C (50 mm × 3.0 mm) under different soaking conditions. The test specimens were stored in distilled water at room temperature for 0, 48 or 168 h followed by a 24 h storage in cell culture medium (DMEM, Dulbecco’s Modified Eagle Medium) under shaking conditions. The DMEM eluates were used undiluted (100%) to determine the monomers by GC-MS. UDMA, urethane dimethacrylate; MMA, methyl methacrylate; BDMA, 1,4-butanediol dimethacrylate; DGEBA, bisphenol A diglycidyl ether; DGEBF, bisphenol F diglycidyl ether. The data are mean values ± SEM of *n* = 3–4 per group.

**Figure 6:**
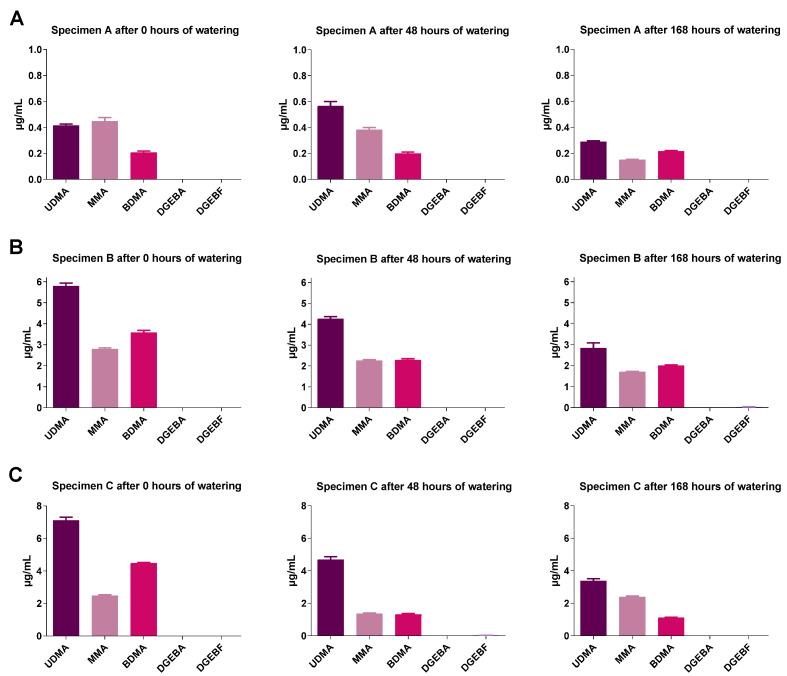
Monomer concentration of Orthocryl^®^ LC specimen extracts of (**A**) test specimens A (15 mm × 0.5 mm), (**B**) test specimens B (50 mm × 1.0 mm), or (**C**) test specimens C (50 mm × 3.0 mm) under different soaking conditions. The test specimens were stored in distilled water at room temperature for 0, 48 or 168 h followed by a 24 h storage in cell culture medium (DMEM, Dulbecco’s Modified Eagle Medium) under shaking conditions. The DMEM eluates were used undiluted (100%) to determine the monomers by GC-MS. UDMA, urethane dimethacrylate; MMA, methyl methacrylate; BDMA, 1,4-butanediol dimethacrylate; DGEBA, bisphenol A diglycidyl ether; DGEBF, bisphenol F diglycidyl ether. The data are mean values ± SEM of *n* = 3–4 per group.

**Figure 7:**
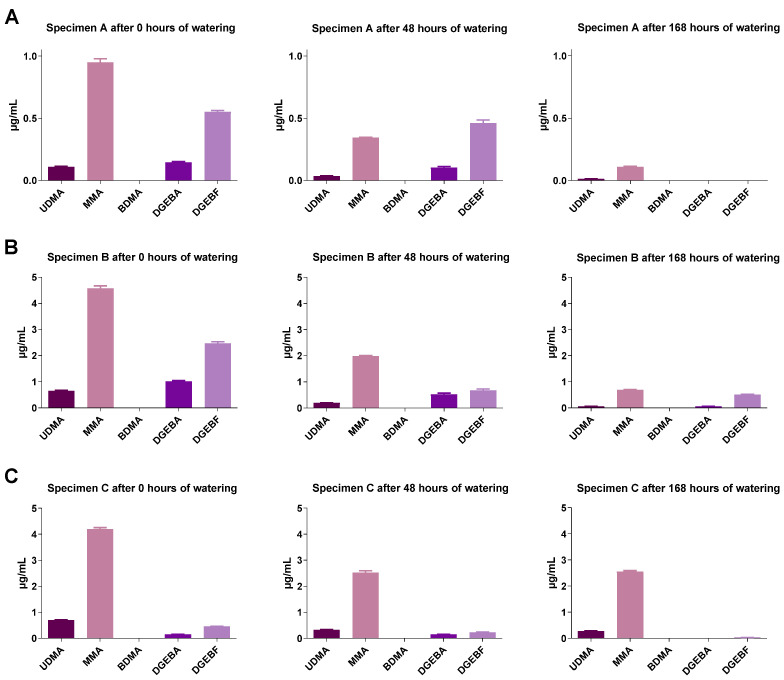
Monomer concentration of Loctite^®^ EA 9483 specimen extracts of (**A**) test specimens A (15 mm × 0.5 mm), (**B**) test specimens B (50 mm × 1.0 mm) or (**C**) test specimens C (50 mm × 3.0 mm) under different soaking conditions. The test specimens were stored in distilled water at room temperature for 0, 48 or 168 h followed by a 24 h storage in cell culture medium (DMEM, Dulbecco’s Modified Eagle Medium) under shaking conditions. The DMEM eluates were used undiluted (100%) to determine the monomers by GC-MS. UDMA, urethane dimethacrylate; MMA, methyl methacrylate; BDMA, 1,4-butanediol dimethacrylate; DGEBA, bisphenol A diglycidyl ether; DGEBF, bisphenol F diglycidyl ether. The data are mean values ± SEM of *n* = 4 per group.

The concentrations of the monomers were measured only in undiluted eluates. The eluates were obtained with constant surface extraction volume ratios. It was found for all geometries that with increasing soaking time the concentrations of the monomers contained in the different plastics decreased strongly (Figure 5, Figure 6 and Figure 7). Test specimen geometry A released lower monomer concentrations than test specimen geometries B and C (by a factor of 5 to 10). Extracts from polypropylene were not studied here, as polypropylene is based on a volatile (gaseous) monomer and is a well-studied non-toxic resin [18].

**Figure 8:**
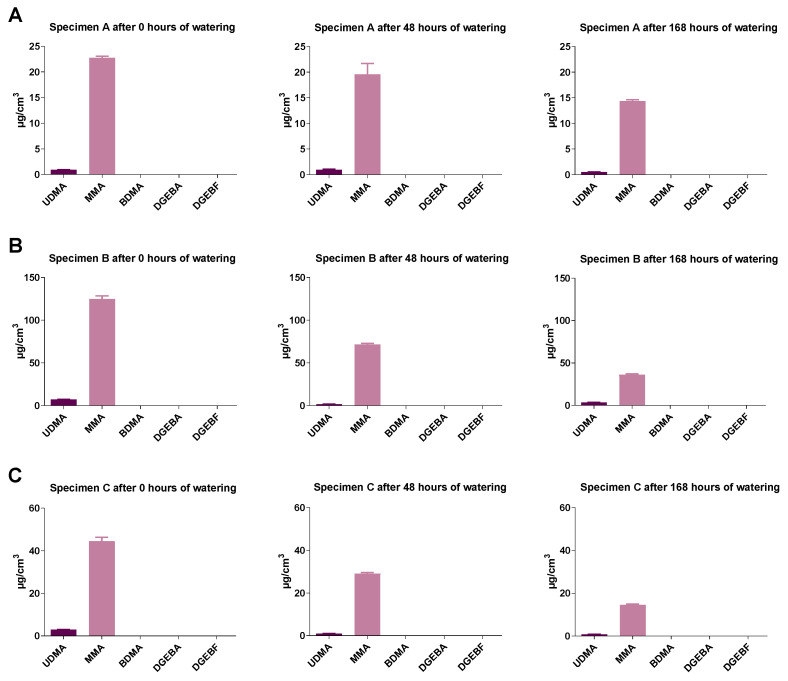
Monomer release from Orthocryl^®^ specimens extracts of (**A**) test specimens A (15 mm × 0.5 mm), (**B**) test specimens B (50 mm × 1.0 mm) or (**C**) test specimens C (50 mm × 3.0 mm) under different soaking conditions. Shown are the total amounts of monomers released in relation to the respective specimen volume. The test specimens were stored in distilled water at room temperature for 0, 48 or 168 h followed by a 24 h storage in cell culture medium (DMEM, Dulbecco’s Modified Eagle Medium) under shaking conditions. The DMEM eluates were used undiluted (100%) to determine the monomers by GC-MS. UDMA, urethane dimethacrylate; MMA, methyl methacrylate; BDMA, 1,4-butanediol dimethacrylate; DGEBA, bisphenol A diglycidyl ether; DGEBF, bisphenol F diglycidyl ether. The data are mean values ± SEM of *n* = 3–4 per group.

In addition to the measured monomer concentrations in the eluates, the eluted monomer amounts were calculated according to the respective specimen volume. As shown in Figure 8 for Orthocryl^®^ specimens, there was a clear trend that soaking resulted in a decrease in monomer concentration in the eluate of all samples tested. Moreover, it became obvious that, relative to the sample volume, parts of geometry B released the largest amounts of monomer (Figure 8, geometry A: MMA, 22.8 ± 0.6 µg/cm^3^; geometry B: MMA, 124.7 ± 6.3 µg/cm^3^; geometry C: MMA, 44.6 ± 3.5 µg/cm^3^). This applied to all plastics (data not shown).

## 3. Discussion

The basis for the in vitro cytotoxicity testing of medical devices is provided by ISO 10993-5:2009 [20]. In this way, certain conditions for in vitro tests can be derived, with which the present work is designed. Thus, eluate direct contact tests can be performed with test specimens of different sizes. These tests are based on qualitative assessment (cell morphology) or quantitative analysis (viability assay). According to the standard, a 30% decrease in viability is indicative of possible cytotoxicity. If necessary, this should be investigated further. However, there are no binding recommendations for the use of specific assays, the possible soaking of the test specimen, or the dimensions of the test specimen in terms of its thickness, shape, or surface-to-volume ratio. Parameters should be adapted to clinical applications, and undiluted eluates should be assayed.

The results of the present study show that the choice of the toxicity test, the geometry of the test specimens, as well as their previous watering time can have a great influence on the evaluation of cytotoxicity.

In the WST-1 assay, which assesses the functionality of the respiratory chain [31], the eluates of the individual plastics and the test sample geometries appear to be very critical in some cases. In particular, significantly decreasing viability was obtained for Orthocryl^®^ LC. The effect of watering the plastics could not always be predicted here. However, the viability values obtained for the WST-1 assay were comparable to those obtained in other studies. For example, Orthocryl^®^ and Orthocryl^®^ LC with geometry A showed similar values to those reported in the literature, although different cell lines and assays were used. Thus, Rose et al. [8] determined viability values of around 80% for Orthocryl^®^ in 50% diluted eluates and between 50 and 80% for light-cured resins in the MTT assay.

In contrast, the Annexin V-FITC-PI assay did not reproduce the extreme decrease in the proportion of viable cells observed in the WST-1 assay. Furthermore, the protective effect of prolonged soaking of the test specimens on their cytotoxic and also proapoptotic effects could also be better elaborated in this assay. Accordingly, almost all significant reductions in viable cell fractions disappeared in the Annexin V-FITC-PI assay when the test specimens was soaked for 168 h. The causes of these discrepant findings are naturally related to the different experimental outcomes. While the WST-1 assay detects cell viability based on mitochondrial activity, the Annexin V-FITC-PI assay uses differences in plasma membrane integrity and permeability to assess whether cells are viable, apoptotic, or necrotic. Regardless, the Annexin V-FITC-PI assay could be an important addition to the investigation and evaluation of specimen with clinically relevant sizes based on in vitro cytotoxicity assays. It is also worth mentioning that in addition to soaking, other measures to reduce cytotoxicity have been reported in the literature, such as treatment with microwaves or ultrasound. However, these have not yet become established in clinical practice or have proved ineffective in some cases [31,32].

Remarkably in our study, samples with geometry B and C gave more critical cytotoxic values in the WST-1 assay than samples with geometry A. The selection of samples with geometry A was based on previous experiments with orthodontic resins, as already described [4,9,13,14,23,24,25,26]. Often small geometries are used that are neither clinically justified nor realistically designed, e.g., cut specimens and small hexagonal geometries [8,13]. However, with respect to our data, testing small, nonclinically adapted test specimen sizes may result in an underestimation of the potential cytotoxic effects of various resins. Furthermore, little or no attention is paid in the literature to the surface-to-volume ratio or the general extraction behavior of plastics. The choice of sample geometry, surface area, volume, or thickness can have a major impact on cytotoxicity and on literature comparisons. Accordingly, the geometries B and C included in our testing correspond more to the clinical situation and show a realistic leakage process and amount of released monomer compared to the sample with geometry A. These observations were confirmed by the results of the Annexin V-FITC-PI test. Specimen C without soaking showed about 40–45% viable cells for Orthocryl^®^ LC 50 and 100, whereby the already cited study by Rose et al. deviated from this due to the smaller specimen geometry they investigated [8].

In the present study, the eluted amount of monomer per sample volume was also measured. This showed that samples with a large surface area generally had the highest amounts of residual monomer in the eluate, although the ratio of surface area to elution volume was constant in our experiments. It should be noted in this context that the surface areas of geometries B and C correspond approximately to the surface area of clinically used devices. Against this background, it can be clearly recommended that samples with a large surface area or a surface area similar to clinical use should be examined for possible cytotoxic effects. A further indication of a possible cytotoxic effect of the released monomers results from a comparison with the literature. Here it could be shown that concentrations of more than 10 µg/mL MMA in the eluate cause changes, such as pseudopodia formation and membrane vesicles in monocytes and granulocytes [27]. Indeed, in the present study, all analyzed eluates of Orthocryl^®^ geometry B and C showed levels above 10 µg/mL MMA. Important in this correlation is a possible different response of A549 cells and the aforementioned cell types. Finally, it should be further investigated why the theoretically larger amount of residual monomer in thicker test specimens with nearly the same surface area in the extraction medium was comparatively small at triplicate thickness (geometry B vs. C). This could be due to an increased diffusion distance in the polymer network or a diffusion barrier due to the dense polymer network. The residual monomer content and also the possible cytotoxic effect of thick samples may be underestimated, but this remains to be clarified. Thus, Löbler et al. also concluded that the evaluation of potential cytotoxicity can already be influenced by the geometry of the test specimens [33]. In this publication, reference is made to ISO 10993-12, which specifies that the surface area to extraction volume ratio for test specimens less than 0.5 mm thick is 6 cm^2^/mL. According to Löbler et al. [33], a change from 6 cm^2^/mL to 3 cm^2^/mL for thicker test specimens, as described in ISO 10993-12:2021 [21], would be equivalent to a 50% extract concentration, ultimately leading to a less pronounced cytotoxic effect.

## 4. Materials and Methods

### 4.1. Specimen Geometries and Polymerization Conditions

Three different circular test specimen geometries were chosen for the tests (each diameter × thickness): A, 15 mm × 0.5 mm; B, 50 mm × 1.0 mm; C, 50 mm × 3.0 mm. Preparation of test specimens was carried out according to the instructions of the manufacturer. In the case of Orthocryl^®^ (DENTAURUM GmbH & Co. KG, Ispringen, Germany), a cold-curing PMMA-based polymer, test specimens were prepared using the sprinkle-on technique. The polymerization conditions were: 2.2 bar in the pressure pot (Polymax 1, Dreve Dentamid GmbH, Unna, Germany) and 40–46 °C for 20 min. Orthocryl^®^ LC (DENTAURUM GmbH & Co. KG, Ispringen, Germany) is a flowable one-component system that was polymerized in a light-curing oven (Individo Light Box, VOCO GmbH, Cuxhaven, Germany). First, a 3-min light cure was performed, then the test specimens were removed from the mold, inverted and cured for a further 10 min. Loctite^®^ EA 9483 (Henkel AG & Co. KGaA, Düsseldorf, Germany) is a two-component epoxy resin (Loctite^®^ 9483 A, Loctite^®^ 9483 B) processed with the manufacturer’s mixing gun (Henkel AG & Co. KGaA, Düsseldorf, Germany). According to the manufacturer, the curing time was 72 h. Polypropylene was prepolymerized and the test specimens were cut from polypropylene sheets (Modulor, Berlin, Germany).

After polymerization, all specimens were rinsed under running water. The oxygen inhibition layers were mechanically removed from all test specimens using a milling cutter for plastics processing (Hager & Meisinger GmbH, Neuss, Germany). The specimen dimensions were then adjusted. Subsequently, the upper side was polished with the KaVo EWL Poliereinheit (KaVo Dental, Biberach an der Riß, Germany) in a three-stage process (Pluradent AG & Co KG, Offenbach, Germany).

### 4.2. Storage of the Specimens and Extraction of the Eluates

The storage of test specimens and the monomer elution procedure were performed according to ISO 10993-5:2009 [20]. Prior to the elution process, samples were either not watered or placed in a volume of 500 mL double distilled water for 48 h or 168 h, respectively. Dulbecco’s Modified Eagle Medium (DMEM, high-glucose, Lonza Group, Basel, Switzerland) without fetal calf serum (FCS) but with antibiotics was used as an elution medium. Elution was performed at 37 °C ± 1 °C in a shaking bath (1.66 Hz, model WNE14, Memmert GmbH + Co. KG, Schwabach, Germany) in a Petri dish (92 × 16 mm, #82.1472, Sarstedt AG & Co, Nürnbrecht, Germany). The cytotoxicity of undiluted extracts was also investigated according to ISO 10993-5:2009 [20]. As proposed, the elution time in DMEM was 24 h. Eluates were freshly prepared before each experiment. The ratio of surface to elution volume of each specimen to the elution medium was 167 µL/cm^2^ as recommended by Löbler et al. [33] resulting in total elution volumes of 0.63 mL, 6.9 mL and 7.33 mL for the three different test specimen geometries.

### 4.3. Cell Culture

A549 human lung carcinoma cells were purchased from DSMZ (Deutsche Sammlung von Mikroorganismen und Zellkulturen GmbH, Braunschweig, Germany). Cells were cultured in DMEM supplemented with 10% heat-inactivated FCS (Pan Biotech, Aidenbach, Germany) 100 U/mL penicillin and 100 µg/mL streptomycin (Thermo Fisher Scientific Inc., Schwerte, Germany) and were grown in a humidified atmosphere (5% CO_2_) at 37 °C in 75 cm^2^ cell culture flasks. Subconfluent cell layers were passaged (1:6) using a standard trypsin/EDTA protocol [34] and the medium was changed every other day.

### 4.4. Eluate Exposure, Cell Viability Assay (WST-1) and Annexin V-FITC-PI Assay

The test specimens were stored in distilled water for different time intervals (0, 48 or 168 h) and then placed in cell culture medium for 24 h. This cell culture medium (eluate) was then used undiluted (100%) or mixed with standard medium (DMEM) at different dilutions (50%, 25%, 12.5%, 6.25%, 3.125%) for the 24 h cultivation of A549 cells, although only in the case of the WST-1 test extract concentrations of less than 25% (i.e., 12.5%, 6.25%, 3.125%) were also included. The cytotoxicity of the eluates was subsequently determined; the quantification of the monomers was limited to the undiluted eluates.

Cell viability was analyzed using the WST-1 assay (Roche Diagnostics, Mannheim, Germany) according to the manufacturer´s instructions. The WST-1 colorimetric assay detects the cleavage of WST-1, a water-soluble tetrazolium salt (4-[3-(4-iodophenyl)-2-(4-nitrophenyl)-2H-5-tetrazolio]-1,3-benzenedisulfonate), to a soluble formazan dye by metabolically active cells and can, therefore, be considered a measure of cell viability. Cells were seeded at 5 × 10^3^ cells per well in 96-well flat-bottom microplates 24 h before exposure to the eluates. After 24 h of incubation of the cells in the extracts, 10 µL of WST-1 reagent was added and after 30 min of incubation, the measurement was performed. Absorbance was measured at 450 nm (reference wavelength 690 nm) using Anthos HT2 (Anthos Labtec Instruments, GmbH, Salzburg, Austria).

The EBioscience™ Annexin V Apoptosis Detection kit (#BMS500FI-300; ThermoFisher Scientific Inc., Schwerte, Gemany) was used for flow cytometry, and analysis was performed using the BD Accuri^TM^ C6 flow cytometer (BD Biosciences, Heidelberg, Germany). To this end, cells were seeded 1 × 10^5^ cells per well in 6-well plates and grown for 24 h. After 24 h of incubation in the extracts, cells were treated according to the manufacturer’s assay protocol. Following washing with phosphate-buffered saline (PBS, Lonza Group, Basel, Switzerland), cells were detached from the wells using Accutase^®^ (Sigma Aldrich, Taufkirchen, Germany). Detached cells were pelleted by centrifugation (120× *g*, 4 °C, 5 min) and washed twice in PBS. Cells were resuspended in 195 μL 1× Binding Buffer and gently vortexed with 5 μL Annexin V-FITC solution. After an incubation period of 10 min, 10 μL propidium iodide solution (20 μg/mL) was added. The following analysis was performed after an incubation period of 5 min in the dark. By analyzing unstained, single-stained and double-stained cells, a correct gating of the cell populations and compensations was ensured. Staurosporin (0.25 µM) was used as a positive control.

### 4.5. Quantification of Eluted Monomers by Gas Chromatography-Mass Spectrometry (GC-MS)

The analysis of monomers was performed according to Reichl et al. [30] with minor modifications. Chromatography was performed on an HP 5890 (Hewlett Packard, Palo Alto, CA, USA) coupled to a 5971 MSD using an HP-1MS column (12 m, inner diameter 0.2 mm, film thickness 0.33 µm) at an injection temperature of 280 °C, a constant oven temperature of 70 °C for 3 min, followed by a temperature gradient of 30 °C/min to 300 °C and a constant temperature of 300 °C for another 6 min, and an interface temperature of 280 °C. Helium was used as the carrier gas at a constant pressure of 24 kPa. The monomers were extracted twice in ethyl acetate and the solutions were evaporated at room temperature in a N_2_ stream to a residual volume of about 150 µL. Monomers were identified based on their retention times and masses using caffeine as an internal standard (Table 1).

In addition to the measured monomer concentrations in the eluates, the eluted monomer amounts were calculated according to the respective specimen volume. UDMA was purchased from Polysciences Europe GmbH (Hirschberg an der Bergstraße, Germany), MMA from DENTAURUM GmbH & Co. KG (Ispringen, Germany) and BDMA, DGEBA and DGEBF from Sigma Aldrich (Taufkirchen, Germany).

### 4.6. Statistical Analysis

Comparisons between groups were carried out using one-way ANOVA with Dunnett’s post hoc test. All statistical analyses were conducted with GraphPad Prism 9.3.0 (GraphPad Software, Inc., San Diego, CA, USA).

## 5. Conclusions

Different cytotoxicity profiles were obtained for the resins tested, with Orthocryl^®^ and polypropylene showing the comparatively best compatibility. However, all tested resins are suitable for use in orthodontics according to the current ISO 10993-5:2009 due to their cytotoxicity tested in vitro. For the group of cold-curing polymers (in this case Orthocryl^®^), there is a clear recommendation from the manufacturers for watering. This also applies to the other polymers used. The extent to which the alternative Loctite^®^ EA 9483 (epoxy resin), which is not yet used in orthodontics, is suitable for clinical use as an orthodontic aid has to be investigated in the future. The main focus should be on the mechanical properties, sensitizing effect and carcinogenicity. In summary, this study highlights the importance of fixed soak times, which should also be reflected in the test protocols proposed by ISO 10993-5:2009. In addition, further evaluation and inclusion of test methods, such as flow cytometry or monomer analysis should be considered.

## Figures and Tables

**Table 1:** Characterization of monomers in GC-MS; the quantification masses are underlined.

Monomer	Masses	Lowest Calibrator
Urethane dimethacrylate (UDMA)	69, 87	0.03 µg/mL
Methyl methacrylate (MMA)	69, 113	0.1 µg/mL
1,4-Butanediol dimethacrylate (BDMA)	69, 87, 140	0.04 µg/mL
Bisphenol A diglycidyl ether (DGEBA)	325, 340	0.03 µg/mL
Bisphenol F diglycidyl ether (DGEBF)	181, 197	0.03 µg/mL
Caffeine (internal standard)	194, 109	Not determined

## Data Availability

The data presented in this study are available on reasonable request from the first author.

## Sample Availability

Samples of the compounds are not available from the authors.
